# Influence of frailty and its interaction with comorbidity on outcomes among total joint replacement

**DOI:** 10.1186/s12891-022-05333-6

**Published:** 2022-04-25

**Authors:** Qiang Lia, Kangxian Li, Qinfeng Yang, Yun Lian, Mingchen Zhao, Zhanjun Shi, Jian Wang, Yang Zhang

**Affiliations:** 1 Division of Orthopaedic Surgery, Department of Orthopaedics, Nanfang Hospital, Southern Medical University, 1838 Guangzhou Avenue, Guangzhou, 510515 Guangdong China; 2 First Affiliation Hospital of Nanchang University, Nanchang City, Jiangxi Province, China; 3 School of Public Health, Peking University, Beijing, China

**Keywords:** Frailty, Comorbidity, Postoperative complications, National inpatient sample, Total joint replacement

## Abstract

**Background:**

Patients with frailty get more and more attention in clinical practice. Yet, no large-scale studies have explored the impact of frailty on the perioperative acute medical and surgical complications following TJA. what is more, comorbid diseases may lead, at least additively, to the development of frailty. There also no studies to find the possible interaction between comorbidity and frailty on the postoperative complications after TJA.

**Methods:**

Discharge data of 2,029,843 patients who underwent TJA from 2005 to 2014 from the National Inpatient Sample (NIS) database, which was analyzed using cross-tabulations and multivariate regression modeling. Frailty was defined based on frailty-defining diagnosis clusters from frailty-defining diagnosis indicator of Johns Hopkins Adjusted Clinical Groups.

**Results:**

Among patients who underwent total joint replacement surgeries, 50,385 (2.5%) were identified as frail. Frailty is highly associated with old age, especially for those over the age of 80, meanwhile females and black races have a high Charlson comorbidity index (CCI) of ≥ 3, together with emergency/urgent admission and teaching hospital. While comorbidity is associated with greater odds of acute medical complications, and frailty has a better predictive effect on in-hospital deaths, acute surgical complications. Furthermore, frailty did not show an enhancement in the predictive power of the Charlson comorbidity score for postoperative complications or in-hospital deaths but postoperative LOS and hospitalization costs.

**Conclusion:**

Frailty can be used to independently predicted postoperative surgical and medical complications, which also has a synergistic interaction with comorbidity for patients who are preparing to undergo TJA.

**Supplementary Information:**

The online version contains supplementary material available at 10.1186/s12891-022-05333-6.

## Introduction

Total joint arthroplasty (TJA) continues being one of the most successful surgeries performed in orthopedic departments [1]. As the average lifespan increases, the need for joint replacement is growing in orthopedics, especially among older adults. The demand for total hip arthroplasty is more than 1 million every year worldwide, and projected to double by 2032, and total knee arthroplasty is expected to increase by 673%, to 3.48 million procedures performed annually by 2030 in the United States, with situations similarly in many countries: Netherlands (297%, 2005–2030),Sweden (163%, 2013–2030),Italy (45%, 2017–2050), the UK( 916%, 2015–2035),Australia, (276%, 2013–2030),Korea (407%, 2001–2010) and Japan (373%,2007–2014) [2–9].

Frailty, a preventable geriatric syndrome, means a non-specific state of decreased ability to respond to acute stress and an increased vulnerability to stressors resulting from an organism’s decline in physiological reserves [10]. Frailty has been recognized as a predictor for adverse events among patients undergoing non-orthopedic surgeries [11–13]. The surgeons also have an increased awareness of frailty. While there are many existing instruments such as clinical frailty scales, FRAIL questionnaire or modified frailty index (mFI) [14, 15]. In this study, frailty was defined by the frailty-defining diagnosis indicator of Johns Hopkins Adjusted Clinical Groups (ACG). There are numerous definitions of frailty. one definition was widely accepted based on the study of Fried et al., which was measured by 5 physical indicators: activity level, gait speed, handgrip strength, involuntary weight loss, and level of exhaustion [16]. However, compared with those numerous definitions of frailty, ACG frailty-defining diagnosis indicator is a more comprehensive definition of frailty, which describes frailty as 70 possible clinical deficits. To adapt to these definitions, ACG frailty-defining diagnosis indicator has been further created through adapting based on the International Classification of Disease, Ninth Revision (ICD-9) by converting 10 clusters of frailty-defining diagnoses into corresponding ICD-9 codes [17].

However, the extent to which frailty impacts perioperative surgical and acute medical complications in TJA has been explored in relatively few large-scale studies [18–20]. In the review of Lemos et al., it was pointed out that some studies have shown that preoperatively frail patients were closely related to perioperative complications and mortality after joint replacement [21]. However, the sample size in the existing studies is far smaler than the sample size of this experiment [22–24]. What is more, in this study, postoperative outcomes were first divided into surgical and acute medical complications. This general classification of the adverse outcomes can help clinicians to conduct appropriate discussions about treatment plans with patients so that they can be used in preoperative counseling before obtaining an informed consent to support surgical decision-making. To be specific, if frail patients are highly associated with postoperative acute medical complications, it means that patients may need to be admitted to the intensive care unit (ICU) department for further treatment after surgeries. On the other hand, if frail patients are highly associated with postoperative surgical complications, clinicians can be reminded to pay attention during the surgery process like not perforating or lacerating the blood vessels, nerves or organs, and strict aseptic techniques should be used. Comorbidity is defined as the concurrent presence of two or more medically diagnosed diseases in the same individual, in which an index disease occurs first. Frail patients often have comorbidities, what is more, comorbid diseases may lead, at least additively, to the development of frailty [13]. Wong et al. reported that among community-dwelling seniors who were frail, 82% had comorbidities [13]. Carrie et al. also found that there was significant interaction between frailty and comorbidities, synergistically increasing the odds of acute medical complications in head and neck cancer (HNCA) surgeries [11]. Although a close relationship exists between frailty and comorbidities, there are no large-scale studies differentiating the effect of frailty and comorbidities on perioperative surgical or medical complications among patients who undergo TJA. Therefore, surgeons or anesthesiologists can assess the preoperative risks of patients more comprehensively, such as considering whether the frailty assessment options should be added to the physical status classes of The American Society of Anaesthesiologists (ASA), which is also taken as a comorbidity index. Due to the different prevention strategies and treatments for patients with frailty and comorbidities, a preoperative medical evaluation of frailty is also of great importance for older patients who undergo joint replacement today [25].

The purpose of this study was to investigate effects of frailty on the perioperative outcomes of joint replacement, and to further investigate the interaction between frailty and comorbidities. We hypothesized that frailty would have a significant influence on both surgical and medical complications of TJA. Additionally, the integration of frailty and comorbidities would be significant for joint replacement surgeries.

## Methods

### Data source

The National (Nationwide) Inpatient Sample (NIS) database was used to identify the information of patients who underwent total joint replacement performed from 2005 to 2014 based on the procedure codes of International Classification of Diseases, Ninth Revision, Clinical Modification (ICD-9-CM).

### Data collection

#### Participant

The inclusion criteria were patients aged 18 years or older who underwent TJA, who were identified according to ICD-9-CM procedural codes of TJA (81.51 and 81.54).

#### Definition of frailty

The definition of frailty was based on 10 sets of frailty-defined diagnoses including the Hopkins Adjusted Clinical Groups (ACG) frailty indicator (a binary variable), and the ICD-9 codes assigned at the time of admission was used (Supplemental Table 1). Frailty-defined diagnoses were different from the diagnosis of comorbidities. Except for dementia, the ICD-9 code 290.0–290.3 defining dementia among the frailty indexes were also part of the definition of dementia in the comorbidity code, but it only accounted for 1.5% of the cases of dementia defined by the comorbidities.

#### Data collection

Data of participants defined above were collected from NIS database from 2005 to 2014. Patients with frailty also defined by ICD-9-CM diagnostic codes as above. We graded comorbidities using the Charlson comorbidity index (CCI), through which the number of pre-existing conditions and their severity were incorporated. In this index, each condition was assigned a score depending on the risk of death associated with that condition [26, 27]. Postoperative complications were divided into medical and surgical complications. Medical complications included acute cardiac events, severe pulmonary edema, acute renal failure, acute hepatic failure, acute cerebrovascular events, sepsis, pneumonia and urinary tract infections. Surgical complications included postoperative infections; non-healing surgical wounds; accidental perforation or laceration of a blood vessel, nerve or organ; a mechanical complication of prosthetic joints; and deep vein thrombosis/pulmonary embolism (DVT/PE) (Supplemental Table 2). All complications had occurred in the hospital and were assigned a corresponding code before discharge.

### Statistical analysis

In this cross-sectional analysis, the analytic cohort consisted of patients with a diagnosis of TJA. Significant differences between the frailty and non-frailty group were determined by a Wilcoxon rank test on continuous data and a chi-square test on categorical data. In addition, the relationship between independent variables and frailty, in-hospital deaths or postoperative complications was analyzed through multivariate logistic regression analyses. Independent variables included age, sex, comorbidities, nature of admission (emergent/urgent, or others), hospital bed size, hospital location (rural or urban) and hospital teaching status. Frailty was also examined as an independent variable in the multivariate logistic regression analyses in which the dependent variable was in-hospital deaths or postoperative complications. Frailty was not examined as an independent variable in the multivariate logistic regression analyses in which the dependent variable was frailty itself [11]. Frailty was also examined as an independent variable in linear regression analyses of the length of stay (LOS) and hospital costs. Generalized linear regression analyses of an increased LOS and hospital costs were used to investigate the interaction between frailty and comorbidities. The non-significant interaction identified in some regression models was removed from the model (Supplemental Fig. 1). Odd ratios (ORs) and 95% of confidence intervals (CIs) were reported for univariate and multivariate analyses.

**Fig.1:**
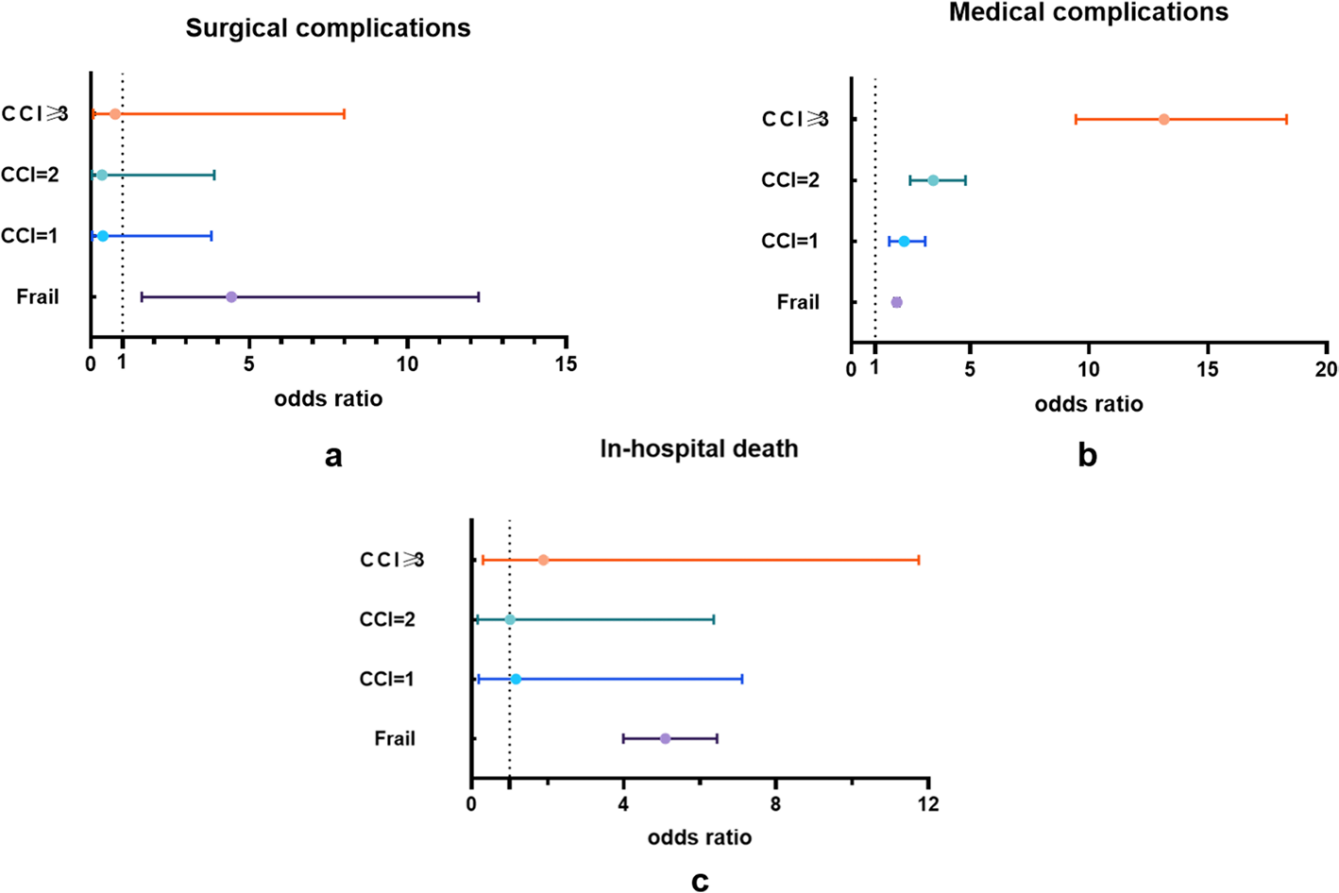
**a** Differences of the Charlson Comorbidity Index and the Frailty on surgical complications of Total Joint Replacement from 2005 to 2014. **b** Differences of the Charlson Comorbidity Index and the Frailty on medical complications of Total Joint Replacement from 2005 to 2014. c. Differences of the Charlson Comorbidity Index and the Frailty on the in-hospital death of Total Joint Replacement from 2005 to 2014

Stata software, R version 3.5.3 (The R Foundation Inc) was used for data analysis. A *P* value < 0.05 with OR and 95% CI was used to determine the statistical significance of the independent variables.

## Results

### Characteristics of frail patients and independent risk factors for frailty

Of the 2,029,843 patients in the NIS database who underwent hip and knee replacement from 2005 to 2014, there were 50,385 patients with frailty (Table 1), the overall incidence rate of which was 2.5%. The incidence rate of patients with TJA over the age of 80 with frailty was two to three times higher than that of non-frails. Most preoperatively frail patients were white, female, with an average age of 74, and were older than those who were not frail. Patients with frailty accounted for 92.4% of those with a CCI ≥ 3 points. The mortality rate among patients with frailty was much higher than that among non-frail patients. The cost of hospitalization was higher for patients with frailty than that for non-frail patients (Table 1). Further, in Table 2, multiple logistic regression analyses show that patients with frailty were significantly associated with being over the age of 80, black races, female gender, a CCI ≥ 3, treatment in hospitals with medium or large bed sizes, teaching hospitals and urban hospitals. Frailty was associated with a decreased odd of emergency/urgent admission and a CCI score of 2.

**Table 1:** Patient Characteristics with frail and Outcomes After THA (2005–2014)

Parameter	Non-Frail	Frail	*P* Value
**Total(*n* = count)**	1,979,458	50,385	**-**
**Total incidence**		2.5%	
**Age (mean ± standard deviation, yrs.)**	67.14 ± 0.03	74.31 ± 0.28	** < **0.0001
**Age group (years)**			< 0.0001
≤ 40	1.4%	0.82%	
40–64	39.3%	20.3%	
65–80	45.7%	42.6%	
≥ 80	13.6%	36.28%	
**Sex**			< 0.0001
Male	38.5%	33.6%	
Female	61.5%	66.4%	
**Race**			< 0.0001
White	71%	74.6%	
Black	5.8%	5.7%	
Hispanic	3.9%	3.9%	
Asian or Pacific Islander	1%	1.1%	
Native American	0.4%	0.36%	
Other	1.8%	1.6%	
**Nature of admission**			< 0.0001
Emergency/Urgent	14.7%	45%	
Elective	85.1%	54.8%	
**Comorbidity**			< 0.0001
**0**	0.93%	4.5%	
**1**	4.4%	1.7%	
**2**	14.8%	5.4%	
** ≥ 3**	79.8%	92.5%	
**Medical complications**			
Acute cardiac event	4.1%	12.8%	< 0.0001
Acute pulmonary edema/failure	0.57%	4.3%	< 0.0001
Acute cerebrovascular event	0.1%	0.3%	< 0.0001
Acute renal failure	2.2%	9.0%	< 0.0001
Acute hepatic failure	0.03%	0.3%	< 0.0001
Pneumonia	0.9%	5.4%	< 0.0001
Urinary tract infection	0.4%	0.6%	< 0.0001
**Surgical complications**			
Postoperative infection	0.1%	0.2%	< 0.0001
Accidental perforation or laceration of blood vessel, nerve, or organ	0.1%	0.1%	< 0.0001
mechanical complication of prosthetic joint	0.6%	1.9%	< 0.0001
DVT/PE^a^	0.7%	1.7%	< 0.0001
**Average cost of hospitalization**	49,402$(47,731,47,831)	69,113$	< 0.0001
**Death rate**	0.3%	2.4%	< 0.0001

^a^*DVT/PE* Deep vein thrombosis/pulmonary embolism

**Table 2:** Multivariate Logistic Regression Analysis of Variables Significantly Associated with Frailty

Variable	OR^a^	95% CI^b^	*P* Value
Age group ≥ 80 years	1.922	[1.88001–1.96560]	< 0.0001
Female	1.0407	[1.01964–1.06216]	0.00013
Black race	1.1063	[1.06376–1.15062]	< 0.0001
Emergency/urgent admission	0.2965	[0.29026–0.30295]	< 0.0001
Charlson comorbidity index 2	0.8129	[0.70114–0.94254]	0.00607
Charlson comorbidity index ≥ 3	1.6231	[1.40687–1.87263]	< 0.0001
Medium bed size	1.3109	[1.27074–1.35230]	< 0.0001
Large bed size	1.2832	[1.24731–1.32016]	< 0.0001
Teaching hospital	1.1	[1.04748–1.09068]	< 0.0001
Urban hospital location	1.1	[1.05354–1.12313]	< 0.0001

^a^OR Odds Ratio

^b^CI Confidence Interval

### Differences between frailty and CCI in in-hospital mortality as well as medical and surgical complications after TJA

Multiple logistic regression analyses comparing the effect of frailty and preoperative comorbidities on mortality, medical and surgical complications are shown in Table 3. After controlling for the effect of all variables, among patients with frailty, being over the age of 80 (OR = 2.97, 95% CI 2.22–3.96, *P* < 0.0001), being in hospitals with a medium (OR = 1.14, 95% CI 1.05–1.23, *P* = 0.0024) or large bed size (OR = 1.21, 95% CI 1.12–1.30, *P* < 0.0001) and being in teaching hospitals (OR = 1.13, 95% CI 1.07–1.19, *P* < 0.0001) were independent factors significantly associated with an increased risk of in-hospital deaths; however, female gender (OR = 0.58, 95% CI 0.55–0.61, *P* < 0.0001), emergency/urgent admission (OR = 0.11, 95% CI 0.11–0.12, *P* < 0.0001) and urban hospital location (OR = 0.91, 95% CI 0.85–0.98, *P* = 0.0129) were significantly associated with decreased odds of mortality. Further, the comorbidity score was not significantly associated with in-hospital deaths. The odds of postoperative surgical complications were significantly higher in hospitals with a medium (OR = 1.14, 95% CI 1.05–1.23, *P* = 0.0024) or large bed size (OR = 1.21, 95% CI 1.12–1.30, *P* < 0.0001), urban hospital locations (OR = 1.63, 95% CI 1.25–2.12, *P* = 0.0003) and patients with frailty (OR = 4.44, 95% CI 1.61–12.24, *P* = 0.0040), whereas older ages (OR = 0.05, 95% CI 0.04–0.07, *P* < 0.0001), female gender (OR = 0.57, 95% CI 1.02–6.63, *P* = 0.0452) and emergency/urgent admission (OR = 0.19, 95% CI 0.17–0.22, *P* < 0.0001) were associated with lower odds of acute surgical complications. The comorbidity score was not significantly associated with surgical complications. Acute medical complications were significantly associated with older ages (OR = 4.15, 95% CI 3.87–4.46, *P* < 0.0001), medium (OR = 1.19, 95% CI 1.17–1.21, *P* < 0.0001), or large-volume hospital care (OR = 1.22, 95% CI 1.22–1.24, *P* < 0.0001), urban hospital location, teaching hospitals (OR = 1.04, 95% CI 1.02–1.06, *P* < 0.0001), frailty (OR = 2.00, 95% CI 1.81–1.90, *P* < 0.0001) and comorbidities (OR = 3.09, 95% CI 1.59–2.22, *P* < 0.0001). The odds of postoperative medical complications were lower among female patients (OR = 0.79, 95% CI 0.79–0.80, < 0.0001) and those with an emergency/urgent admission (OR = 0.34, 95% CI 0.34–0.34, *P* < 0.0001). Frailty was associated with lower odds of acute medical complications than that for comorbidities.

**Table 3:** Multivariate Logistic Regression Analysis of Variables Significantly Associated with Risk of In-Hospital Death and Postoperative Complications

Variable	OR^a^	95% CI^b^	*P* Value
**In-hospital death**			
Age group ≥ 80 years	2.97051	[2.22332,3.9688]	< 0.0001
Female	0.58008	[0.55220–0.6094]	< 0.0001
Emergency/urgent admission	0.11309	[0.10627–0.1203]	< 0.0001
Medium bed size	1.13505	[1.04606–1.2316]	0.00236
Large bed size	1.20576	[1.11979–1.2983]	< 0.0001
Teaching hospital	1.12946	[1.07281–1.1891]	< 0.0001
Urban hospital location	0.91320	[0.85014–0.9809]	0.01288
Frail	5.09046	[3.9917–6.44707]	< 0.0001
Charlson comorbidity index = 1	1.16483	[0.1909–7.10789]	0.86867
Charlson comorbidity index = 2	1.01096	[0.1607–6.35993]	0.99073
Charlson comorbidity index ≥ 3	1.88958	[0.3041–11.74262]	0.49478
**Surgical complications**			
40 to 64 years	0.17026	[0.13909–0.20842]	< 0.0001
65 to 80 years	0.07460	[0.05996–0.09281]	< 0.0001
> 80 years	0.05248	[0.04036–0.06826]	< 0.0001
Female	0.57444	[0.50722–0.65057]	< 0.0001
Emergency/urgent admission	0.19200	[0.16757–0.21999]	< 0.0001
Medium bed size	1.13505	[1.04606–1.2316]	0.00236
Large bed size	1.20576	[1.11979–1.2983]	< 0.0001
Teaching hospital	2.11839	[1.85358–2.42104]	< 0.0001
Urban hospital location	1.63107	[1.25291–2.12337]	0.00028
Frail	4.43661	[1.60858–12.23655]	0.00400
Charlson comorbidity index = 1	0.37590	[0.03719–3.79894]	0.40707
Charlson comorbidity index = 2	0.34613	[0.03080–3.88964]	0.39004
Charlson comorbidity index ≥ 3	0.76419	[0.07303–7.99691]	0.82237
**Medical complications**			
40 to 64 years	1.64373	[1.53057–1.76525]	< 0.0001
65 to 80 years	2.09409	[1.95107–2.24759]	< 0.0001
> 80 years	4.15499	[3.86966–4.46135]	< 0.0001
Female	0.79571	[0.78688–0.80465]	< 0.0001
Emergency/urgent admission	0.33972	[0.33555–0.34393]	< 0.0001
Medium bed size	1.18791	[1.16748–1.20869]	< 0.0001
Large bed size	1.22394	[1.20499–1.24318]	< 0.0001
Teaching hospital	1.14781	[1.13486–1.16091]	< 0.0001
Urban hospital location	1.04192	[1.02415–1.06001]	< 0.0001
Frail	2.00216	[1.81372–1.90562]	< 0.0001
Charlson comorbidity index = 1	3.09695	[1.58599–2.21624]	< 0.0001
Charlson comorbidity index = 2	4.79525	[2.46746,3.43978]	< 0.0001
Charlson comorbidity index ≥ 3	18.30816	[9.44622, 13.15078]	< 0.0001

^a^OR Odds Ratio

^b^CI Confidence Interval

**Table 4:** Generalized Linear Regression Analysis of Length of Stay and Hospital Costs

Variable	OR^a^	95% CI^b^	*P* Value
**Length of stay (days)**			
40 to 64 years	-0.17228	[-0.20180– -0.14275]	< 0.0001
65 to 80 years	-0.07707	[-0.10659– -0.04755]	< 0.0001
> 80 years	0.45668	[0.42602–0.48733]	< 0.0001
Female	-1.95904	[-1.96905– -1.94903]	< 0.0001
Emergency/urgent admission	0.03105	[0.02409–0.03801]	< 0.0001
Medium bed size	0.10855	[0.09828–0.11881]	< 0.0001
Large bed size	0.22233	[0.21324–0.23142]	< 0.0001
Teaching hospital	0.12199	[0.11495–0.12904]	< 0.0001
Urban hospital location	0.00993	[-0.00096–0.02082]	< 0.0001
Non-Frail, CCI^c^ = 1	0.18586	[0.11042–0.26129	< 0.0001
Non-Frail, CCI = 2	0.24974	[0.17365–0.32584]	< 0.0001
Non-Frail, CCI ≥ 3	0.44118	[0.36514– 0.51723]	< 0.0001
Frail, CCI = 0	1.53533	[1.09748–1.97318]	< 0.0001
Frail, CCI = 1	0.57325	[0.38423–0.76227]	< 0.0001
Frail, CCI = 2	0.58512	[0.46956–0.70068]	< 0.0001
Frail, CCI ≥ 3	1.31211	[1.23151–1.39271]	< 0.0001
**Cost of hospitalization (Dollars)**			
40 to 64 years	$-5122.97	[-5509.2– -4736.8]	< 0.0001
65 to 80 years	$-6494.01	[-6880.1– -6107.9]	< 0.0001
> 80 years	$-6415.72	[-6816.7– -6014.7]	< 0.0001
Female	$-7194.00	[-7325.0– -7063.0]	< 0.0001
Emergency/urgent admission	$-1626.37	[-1717.7– -1535.0]	< 0.0001
Medium bed size	$2816.81	[2681.8–2951.8]	< 0.0001
Large bed size	$3434.61	[3315.5–3553.7]	< 0.0001
Teaching hospital	$-1701.21	[-1793.7–-1608.7]	< 0.0001
Urban hospital location	$11,664.11	[11522.2–11,806.0]	< 0.0001
Non-Frail, CCI = 1	$3151.7	[1899.9–4403.4]	< 0.0001
Non = Frail, CCI = 2	$3630.6	[2367.9–4893.4]	< 0.0001
Non-Frail, CCI ≥ 3	$5443.3	[4181.4–6705.2]	< 0.0001
Frail, CCI = 0	$26,426.7	[19202.9–33,650.6]	< 0.0001
Frail, CCI = 1	$13,345.7	[10213.9–16,477.5]	< 0.0001
Frail, CCI = 2	$13,885.7	[11966.2–15,805.1]	< 0.0001
Frail, CCI ≥ 3	$16,748.2	[15409.8–18,086.6]	< 0.0001

*CCI* Charlson comorbidity index

^a^*OR* Odds Ratio

^b^CI: Confidence Interval

Compared with comorbidities, the correlation between frailty and hospital mortality or surgical complications had a greater significance (Fig. 1a and c). In contrast, fragility was less relevant for medical complications than it was for comorbidities (Fig. 1b). The interaction between frailty and comorbidities was not substantial regarding mortality and internal or surgical complications.

### Interaction of frailty and CCI on LOS and the cost of hospitalization after TJA

**Fig. 2:**
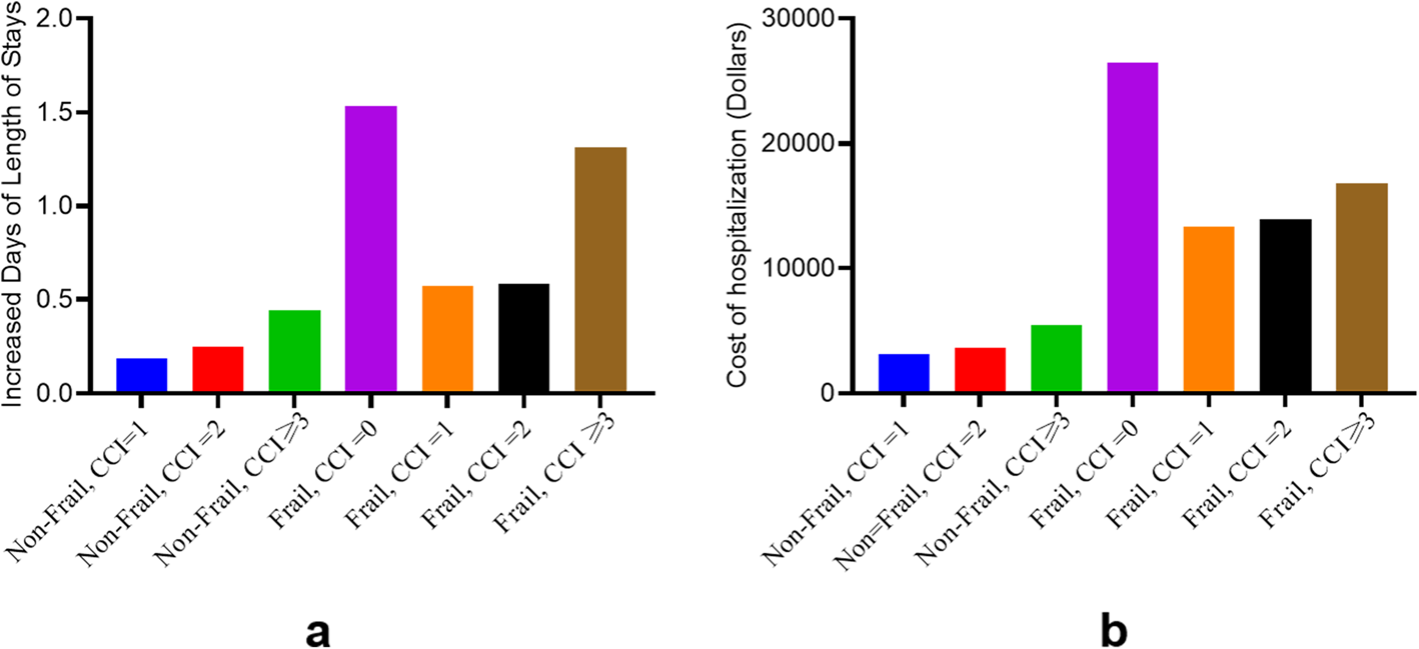
**a** comparing with the combined indictor (Non-frail, CCI = 0), the increased days of LOS of patients undergoing TJA. **b** comparing with the combined indictor (Non-frail, CCI = 0), the increased days of LOS of patients undergoing TJA

A linear multiple regression analysis of the independent variables associated with hospitalization time or costs is shown in Table 4. The result shows that for patients who were older than 80, female gender, being in hospitals with a large bed size, being in teaching hospitals and urban hospital locations were significantly associated with a longer hospitalization time. The interaction between frailty and comorbidities was significant for the length of hospital stay (Fig. 2a). Hospital costs were significantly associated with medium and large bed sizes and urban locations. The interaction between frailty and comorbidities was significant for the cost of hospitalization (Fig. 2b). Furthermore, frailty was a good predictor for the increased LOS and hospitalization costs (Supplemental Table 3).

## Discussion

This study show that frailty is an independent risk factor for postoperative acute medical and surgical complications, while comorbidities only act as an independent risk factor for acute medical diseases. What is more, despite the close association between comorbidities and frailty, comorbidities still did not show a significant interaction with frailty in postoperative acute medical complications of the patients undergoing TJA, which is not consistent with study hypotheses. Differently, as expected, frailty and comorbidities interacted significantly with respect to length of stay and hospital costs.

In this study, the frailty rate of joint replacement was only 2.5%, which was consistent with the results of McIsaac et al. However, the incidence rate was also far lower than that for other surgical operations, which ranged from 8–28%, possibly because the high rates of frailty comprised only older adults [11].

It was found in our study that frailty was significantly associated with advanced comorbidities (CCI ≥ 3), which reflects that among patients undergoing TJA, those who are frail before surgeries are easily combined with comorbidities, which may also be related to the comorbidities as a major cause of frailty. We further found the interaction between frailty and comorbidities not significant, suggesting that comorbidities did not affect the state of frailty during the short-term perioperative period. This means that in the preoperative assessment of patients who are frail before surgeries, there is no need to be afraid whether the comorbidities may increase the risk of postoperative frailty in-hospital deaths or postoperative surgical complications. This paper shows that frailty does not affect the impact of comorbidities on the perioperative period of TJA as it does on HNCA surgeries [11]. It may be because that patients with cancers are of poorer health than those with TJA, and the interaction between frailty and preoperative comorbidities is more likely to be amplified in such surgeries. Although the interaction between comorbidities and frailty in TJA is not yet obvious in the perioperative period, further studies are needed for the long-term postoperative complications.

There was a synergistic interaction between frailty and comorbidities on the length of stay and hospitalization costs. Generally speaking, the costs and LOS of a non-frail patient are far less than that of those with frailty (Fig. 2a and b). Interestingly, the combined predictor, frailty and CCI = 0 had a greater influence on the length of stay and hospitalization costs than the other predictor, which was CCI ≠ 0, suggesting that for people without comorbidities, frailty was easy to be neglected by doctors, which would make patients cost more in hospital.

What is more, previous studies did not include such large-scale data, and their instruments for frailty measurement were not ACG frailty-defining diagnosis indicator, meanwhile a definition of frail led to more comprehensive diagnoses in this study, which might also be a reason for the large amount of data extracted.

This study has some limitations due to the limitations of the NIS database. First of all, as in any large database, there may be discrepancies or misclassification in coding and documentation that lead to an erroneous estimation of frailty. Secondly, because the long-term complications or re-admission indicators were not included in the database, the delayed onset in some cases of frailty or postoperative complications might also result in statistical deviations since the NIS database could only be used to record complications during hospitalization. In addition, the period of data we could choose to observe was limited by the ICD-9-CM system, which was used to adapt for ACG frailty-defining diagnosis indicator. Moreover, the specific mechanism of the effect of frailty on postoperative complications associated with TJA required further investigation. Frailty was not stratified according to different severities such as the Clinical Frailty Scale because of the limitations of the database.

Nevertheless, this study demonstrates the importance of frailty in joint replacement surgeries. Frailty and comorbidities are often present in patients concurrently; however, frailty is independent of comorbidities, which has an impact on the postoperative complications associated with joint replacement. From the perspective of the synergistic interaction between frailty and comorbidities on hospitalization costs and the length of stay, frailty is a factor that requires consideration for joint replacement.

When doctors understand the contribution and interaction of frailty and comorbidities, they can optimize the patient’s status before or during surgeres. Regarding patients with multiple comorbidities, doctors should consider multi-drug therapies to prevent acute medical complications or balance the advantages and disadvantages of the risks caused by comorbidities as well as the benefits after TJA [28, 29].

what is more, family support and physical assistance are also essential for managing comorbidities. Consideration and management of comorbidities before TJA may prevent some comorbidities from becoming unstable, which could further prevent the occurrence of the acute medical complications following TJA.

In this study, frailty had shown a significant impact on perioperative medical and surgical complications and mortality in the joint replacement population. If frailty can be optimization, the incidence of perioperative complications or the death rate may can be reduced following TJA. Here are some suggestions to help relieve frailty. According to many studies, exercise is an essential part of the treatment for frailty [30–33]. Since sedentary lifestyles increased odds of developing frailty, frailty people are recommended to achieve moderate-intensity aerobic exercise more than 150 min/week, such as walking [30, 32]. If the frail patient has difficulty walking due to arthritis, some light houseworks such as doing laundry may also be considered [32]. During the perioperative period, progressive strength training before surgery has been deemed the key exercise for patients with frailty [34]. In addition, for preoperative optimization of frailty, guidelines issued by Centre for Perioperative Care (CPOC) in collaboration with the British Society of Geriatrics in recent years suggest perioperative preventive management of frail patients requiring elective and emergency surgery [35]. This guideline highlights the importance of multidisciplinary collaboration between surgeons, anesthesiologists, and geriatricians to optimize coexisting medical conditions, medications, physical activity, and geriatric syndromes such as malnutrition, sarcopenia and cognitive impairment. This guideline recommendation is already applied in some units in the UK. Specifically, for sarcopenia, preoperative progressive strength training is considered a key exercise in frail patients [34]. For malnutrition, it can be alleviated by nutritional conditioning with targeted protein or carbohydrate loads [36]. For cognitive impairment, patients receiving TJA have been reported to be at high risk for postoperative delirium (POD). To reduce the chance of POD, close attention to intraoperative control of blood pressure, depth of anaesthesia, and temperature should be performed [37]. The above guidelines further mention that management of frail patients before and during surgery. it is possible to consider changing the order of surgical patients before surgery to reduce the hunger time of frail patients. In addition, normal body temperature should be maintained in frail patients during surgery—frail patients may have impaired thermoregulation. Invasive temperature monitoring can be considered if necessary. Also, due to sarcopenia and possibly a higher percentage of adipose tissue in frail patients, these would allow lipophilic drugs to have a larger volume of distribution and possibly longer duration of action, while hydrophilic drugs would have a higher peak plasma concentration. Therefore, the dose of anesthetics should be reconsidered.

## Conclusion

For patients with frailty before TJA, clinicians need to pay special attention to them and prevent the occurrence of acute medical complications in the perioperative period after surgeries. if necessary, frail patients after TJA should be sent to the ICU Department to strengthen management of acute medical complications. Frail patients also have perioperative surgical complications in the perioperative period, such as neurovascular injuries. What is more, compared with CCI, frailty has a greater influence on surgical complications, which might be the factor to increase the in-hospital death rate among patients with frailty who undergo TJA. So, patients with frailty need to be paid more attention to in the surgery process of TJA to prevent surgical complications and in-hospital deaths. Interestingly, many related studies have shown that frailty and preoperative comorbidities are closely related, yet frailty and preoperative comorbidity-related indicators (CCI) in this study were not shown with a significant interaction in medical medicines, which made it questionable whether preoperative comorbidities or frailty should be taken as an overall evaluation index. More studies should be made to judge the interaction between comorbidities and frailty for TJA in the future.

## Supplementary Information

**Additional file 1:** **Supplemental Table 1.** ICD-9 Diagnosis Codes for Frailty. **Supplemental Table 2. **ICD-9 Diagnosis Codes for Acute Postoperative Complications. **Supplemental Table 3. **Generalized Linear Regression Analysis of Length of Stay and Hospital Costs. 

**Additional file 2:** **Supplementary figure 1. **Flow diagram of the data collection and analysis.

## Data Availability

These data are easily available from the Agency for Healthcare Research and Quality (AHRQ’s) “Healthcare Cost and Utilization Project (HCUP)” and can be obtained after completing an on-line Data Use Agreement training session and signing a Data Use Agreement. The contact information for requesting the data is as follows: HCUP Central Distributor. Phone: (866) 556–4287 (toll-free). Fax: (866) 792–5313. E-mail: HCUPDistributor@ahrq.gov.

## Abbreviations

TJA: Total joint replacement
NIS: National Inpatient Sample
LOS: Length Of Stay
ICD-9-CM: International Classification of Diseases, Ninth Revision, Clinical Modification
CCI: Charlson comorbidity index

## References

[1] Sloan M, Premkumar A, Sheth NP (2018). Projected volume of primary total joint arthroplasty in the US, 2014 to 2030. JBJS.

[2] Halpern M, Kurtz S, Lau E, Mowat F, Ong K (2007). Projections of primary and revision hip and knee arthroplasty in the United States from 2005 to 2030. J Bone Surg.

[3] Pivec R, Johnson AJ, Mears SC, Mont MA (2012). Hip arthroplasty. Lancet.

[4] Otten R, van Roermund PM, Picavet H (2010). Trends in the number of knee and hip arthroplasties: considerably more knee and hip prostheses due to osteoarthritis in 2030. Ned Tijdschr Geneeskd.

[5] Nemes S, Rolfson O, W-Dahl A, Garellick G, Sundberg M, Kärrholm J, Robertsson O (2015). Historical view and future demand for knee arthroplasty in Sweden. Acta Orthop.

[6] Romanini E, Decarolis F, Luzi I, Zanoli G, Venosa M, Laricchiuta P, Carrani E, Torre M (2019). Total knee arthroplasty in Italy: reflections from the last fifteen years and projections for the next thirty. Int Orthop.

[7] Culliford D, Maskell J, Judge A, Cooper C, Prieto-Alhambra D, Arden N, Group, C. S. (2015). Future projections of total hip and knee arthroplasty in the UK: results from the UK Clinical Practice Research Datalink. Osteoarthritis Cartilage.

[8] Ackerman IN, Bohensky MA, Zomer E, Tacey M, Gorelik A, Brand CA, De Steiger R (2019). The projected burden of primary total knee and hip replacement for osteoarthritis in Australia to the year 2030. BMC Musculoskelet Disord.

[9] Koh IJ, Kim TK, Chang CB, Cho HJ, In Y (2013). Trends in use of total knee arthroplasty in Korea from 2001 to 2010. Clin Orthop Related Res.

[10] Shah AK, Celestin J, Parks ML, Levy RN (2004). Long-term results of total joint arthroplasty in elderly patients who are frail. Clin Orthop Related Res.

[11] Nieman CL, Pitman KT, Tufaro AP, Eisele DW, Frick KD, Gourin CG (2018). The effect of frailty on short-term outcomes after head and neck cancer surgery. Laryngoscope.

[12] Makary MA, Segev DL, Pronovost PJ, Syin D, Bandeen-Roche K, Patel P, Takenaga R, Devgan L, Holzmueller CG, Tian J (2010). Frailty as a predictor of surgical outcomes in older patients. J Am Coll Surg.

[13] Murad K, Kitzman DW (2012). Frailty and multiple comorbidities in the elderly patient with heart failure: implications for management. Heart Fail Rev.

[14] Buta BJ, Walston JD, Godino JG, Park M, Kalyani RR, Xue Q-L, Bandeen-Roche K, Varadhan R (2016). Frailty assessment instruments: systematic characterization of the uses and contexts of highly-cited instruments. Ageing Res Rev.

[15] Dent E, Martin FC, Bergman H, Woo J, Romero-Ortuno R, Walston JD (2019). Management of frailty: opportunities, challenges, and future directions. Lancet.

[16] Fried LP, Tangen CM, Walston J, Newman AB, Hirsch C, Gottdiener J, Seeman T, Tracy R, Kop WJ, Burke G (2001). Frailty in older adults: evidence for a phenotype. J Gerontol A Biol Sci Med Sci.

[17] Michel J, Goel AN, Golla V, Lenis AT, Johnson DC, Chamie K, Litwin MS (2019). Predicting short-term outcomes after radical cystectomy based on frailty. Urology.

[18] McIsaac D, Beaule P, Bryson G, Van Walraven C (2016). The impact of frailty on outcomes and healthcare resource usage after total joint arthroplasty: a population-based cohort study. Bone Joint J.

[19] Dayama A, Olorunfemi O, Greenbaum S, Stone ME, McNelis J (2016). Impact of frailty on outcomes in geriatric femoral neck fracture management: an analysis of national surgical quality improvement program dataset. Int J Surg.

[20] Wang HT, Fafard J, Ahern S, Vendittoli P-A, Hebert P (2018). Frailty as a predictor of hospital length of stay after elective total joint replacements in elderly patients. BMC Musculoskelet Disord.

[21] Lemos JL, Welch JM, Xiao M, Shapiro LM, Adeli E, Kamal RN (2021). Is frailty associated with adverse outcomes after orthopaedic surgery?: a systematic review and assessment of definitions. JBJS Rev.

[22] Meyer M, Parik L, Leiß F, Renkawitz T, Grifka J, Weber M (2020). Hospital frailty risk score predicts adverse events in primary total hip and knee arthroplasty. J Arthroplasty.

[23] Johnson RL, Abdel MP, Frank RD, Chamberlain AM, Habermann EB, Mantilla CB (2019). Impact of frailty on outcomes after primary and revision total hip arthroplasty. J Arthroplasty.

[24] Traven SA, Reeves RA, Sekar MG, Slone HS, Walton ZJ (2019). New 5-factor modified frailty index predicts morbidity and mortality in primary hip and knee arthroplasty. J Arthroplasty.

[25] Ambagtsheer RC, Beilby JJ, Visvanathan R, Dent E, Yu S, Braunack-Mayer AJ (2019). Should we screen for frailty in primary care settings? A fresh perspective on the frailty evidence base: a narrative review. Prev Med.

[26] Charlson ME, Pompei P, Ales KL, MacKenzie CR (1987). A new method of classifying prognostic comorbidity in longitudinal studies: development and validation. J Chronic Dis..

[27] O’Connell R, Lim L-Y (2000). Utility of the Charlson comorbidity index computed from routinely collected hospital discharge diagnosis codes. Methods Inf Med.

[28] Algahtani R, Merenda A (2021). Multimorbidity and critical care neurosurgery: minimizing major perioperative cardiopulmonary complications. Neurocrit Care.

[29] Williams A, Dunning T, Manias E (2007). Continuity of care and general wellbeing of patients with comorbidities requiring joint replacement. J Adv Nurs.

[30] McPhee JS, French DP, Jackson D, Nazroo J, Pendleton N, Degens H (2016). Physical activity in older age: perspectives for healthy ageing and frailty. Biogerontology.

[31] Ahmed HM, Babakir-Mina M (2021). Population-level interventions based on walking and cycling as a means to increase physical activity. Phys Act Health..

[32] Peterson MJ, Giuliani C, Morey MC, Pieper CF, Evenson KR, Mercer V, Cohen HJ, Visser M, Brach JS, Kritchevsky SB (2009). Physical activity as a preventative factor for frailty: the health, aging, and body composition study. J Gerontol A Biol Sci Med Sci.

[33] Van Der Bij AK, Laurant MG, Wensing M (2002). Effectiveness of physical activity interventions for older adults: a review. Am J Prev Med.

[34] Milte R, Crotty M (2014). Musculoskeletal health, frailty and functional decline. Best Pract Res Clin Rheumatol.

[35] Kane AD, Knight J, Ayyash R (2022). The perioperative management of frailty in patients presenting for vascular surgery. Anaesth Intensive Care Med..

[36] Stojanovic MD, Markovic DZ, Vukovic AZ, Dinic VD, Nikolic AN, Maricic TG, Janković RJ (2018). Enhanced recovery after vascular surgery. Front Med.

[37] Chan MT, Cheng BC, Lee TM, Gin T, Group, C. T. (2013). BIS-guided anesthesia decreases postoperative delirium and cognitive decline. J Neurosurg Anesthesiol.

