# Correction: Influence of frailty and its interaction with comorbidity on outcomes among total joint replacement

**DOI:** 10.1186/s12891-022-05425-3

**Published:** 2022-05-19

**Authors:** Qiang Lian, Kangxian Li, Qinfeng Yang, Yun Lian, Mingchen Zhao, Zhanjun Shi, Jian Wang, Yang Zhang

**Affiliations:** 1 Division of Orthopaedic Surgery, Department of Orthopaedics, Nanfang Hospital, Southern Medical University, 1838 Guangzhou Avenue, Guangzhou, 510515 Guangdong China; 2 First Affiliation Hospital of Nanchang University, Nanchang City, Jiangxi Province China; 3 School of Public Health, Peking University, Beijing, China

**Correction: BMC Musculoskelet Disord 23, 384 (2022)**

**https://doi.org/10.1186/s12891-022-05333-6**

Following the publication of the original article [1] the authors reported that spelling of Dr. Qiang Lian's last name was "Lia" rather than "Lian".

The original article [1] has been updated.
