# Supplementary material for: Influence of frailty and its interaction with comorbidity on outcomes among total joint replacement
Source: BMC Musculoskelet Disord. 2022 Apr 25;23:384. doi: 10.1186/s12891-022-05333-6 (PMC9040243; doi:10.1186/s12891-022-05333-6)
Supplement: Supplementary file 1 — Additional file 1: Supplemental Table 1. ICD-9 Diagnosis Codes for Frailty. Supplemental Table 2. ICD-9 Diagnosis Codes for Acute Postoperative Complications. Supplemental Table 3. Generalized Linear Regression Analysis of Length of Stay and Hospital Costs. [file 12891_2022_5333_MOESM1_ESM.docx]

**Supplemental Table 1： ICD-9 Diagnosis Codes for Frailty**

| Frailty-Defining Diagnoses in the Johns Hopkins ACG^a^ Frailty Indicator with corresponding ICD-9 Codes.  **Variable Diagnoses ICD9** | | |
| --- | --- | --- |
| Malnutrition | Nutritional marasmus  Other severe protein-calorie malnutrition | 261,262,263.8, 263.9, V77.2 |
| Dementia | Senile dementia with delusional or depressive feature  Senile dementia with delirium | 290.20,290.21,290.3 |
| Severe vision impairment | Profound impairment, both eyes Moderate or severe impairment, better eye/lesser eye: profound | 369.0,369.00,369.01, 369.03,369.04,369.06, 369.07, 369.08 |
| Decubitus ulcer | Decubitus ulcer | 707.0,707.00,707.01,707.02,707.03,707.04, 707.05,707.06,707.07,707.09,707.20,707.21, 707.22,707.23,707.24, 707.25 |
| Urinary incontinence | Incontinence without sensory awareness Continuous leakage | 788.34, 788.37 |
| Weight loss | Abnormal loss of weight and underweight Feeding difficulties and mismanagement | 783.2, 783.21, 783.22, 783.3 |
| Fecal incontinence | Incontinence of feces | 787.6 |
| Social support needs | Lack of housing  Inadequate housing  Inadequate material resources | V60.0, V60.1, V60.2, |
| Difficulty in walking | Difficulty in walking  Abnormality of gait | 719.7, 781.2 |
| Fall | Fall on stairs or steps  Fall from wheelchair | E880, E880.0, E880.1, E880.9, E884.3 |

^a^ACG: Adjusted Clinical Groups; ^b^ICD-9: International Classification of Disease, Ninth Revision.

**Supplemental Table 2： ICD-9 Diagnosis Codes for Acute Postoperative Complications**

| **Variable** | **ICD-9 Code** |
| --- | --- |
| **Surgical complications** | |
| Shock | 998.0 |
| Hemorrhage, hematoma, or seroma | 998.1, 998.11, 998.12, 998.13 |
| Accidental perforation or laceration of blood vessel, nerve, or organ | 998.2 |
| Wound dehiscence | 998.3, 998.30, 998.31, 998.32, 998.33 |
| Postoperative infection | 711，7110，71100，71105，7116，71160，7119，71190，71195，730，73000，73005，7301，73010，73015，7302，73025，7309，73090，73095 |
| Fracture of neck, shaft, or unspecified - femur | 820，8200，82001，82003，82009，8201，82010，82011，82012，82013，82019，8202，82020，82021，82022，8203，82030，8208，8209，8210，82100，82101，8211，82110，82111 |
| Non-healing surgical wound | 998.83 |
| Other unspecified procedural complications | 998.8, 998.81, 998.89, 998.9 |
| DVT/PE | 41511，41519，45340，45341，45342 |
| Mechanical complication of prosthetic joint | 99640-99647，99649 |
|  |  |
| **Medical complications** | |
| Acute cardiac event | 410.0-410.9, 411.1, 411.8, 415.0, 420.0, 420.9, 421.0, 421.1, 421.9, 422.0, 422.9, 427.0-427.5, 428.0-428.9 |
| Acute pulmonary edema/failure | 518.4, 518.81, 518.82, 518.84 |
| Acute cerebrovascular event | 997.00, 997.01, 997.02, 997.09 |
| Acute renal failure | 584.5-584.9 |
| Acute hepatic failure | 570 |
| Pneumonia | 480,480.0, 480.1, 480.2, 480.3, 480.8, 480.9, 481, 482, 482.0, 482.1, 482.3, 482.30, 482.31, 482.32, 482.39, 482.40, 482.41, 482.42, 482.49, 482.8, 482.81, 482,82, 482.83, 482.84, 482.89, 482.9, 483, 483.1, 483.8, 484, 484.1, 484.3, 484.5, 484.6, 484.7, 484.8, 485, 487.0, V12.61, 507.0, 514, 518.4, 518.5, 516, 516.8, 997.31 |
| Sepsis | 995.9, 038.0-038.4, 999.3 |
| Urinary tract infection | 599.0, 996.64, 996.31, V13.02 |

^a^DVT/PE: Deep vein thrombosis/pulmonary embolism

| **Supplemental Table 3. Generalized Linear Regression Analysis of Length of Stay and Hospital Costs** | | | |
| --- | --- | --- | --- |
| **Variable** | **OR^a^** | **95% CI^b^** | **P Value** |
| **Length of stay (days)** |  |  |  |
| Frail | 0.81751 | [0.79195–0.84307] | <0.0001 |
| Charlson comorbidity index=1 | 0.17376 | [0.09847–0.24904] | <0.0001 |
| Charlson comorbidity index=2 | 0.23732 | [0.16137–0.31328] | <0.0001 |
| Charlson comorbidity index≥3 | 0.43317 | [0.35726–0.50908] | <0.0001 |
| **Cost of hospitalization (US Dollars)** |  |  | <0.0001 |
| Frail | 11243.9 | [10817.5–11670.3] | <0.0001 |
| Charlson comorbidity index=1 | 2977.6 | [1728.5–4226.7] | <0.0001 |
| Charlson comorbidity index=2 | 3457.3 | [2197.0–4717.6] | <0.0001 |
| Charlson comorbidity index≥3 | 5278.4 | [4019.0–6537.9] | <0.0001 |
