# Supplementary figures and images for: Influence of frailty and its interaction with comorbidity on outcomes among total joint replacement
Source: BMC Musculoskelet Disord. 2022 Apr 25;23:384. doi: 10.1186/s12891-022-05333-6 (PMC9040243; doi:10.1186/s12891-022-05333-6)

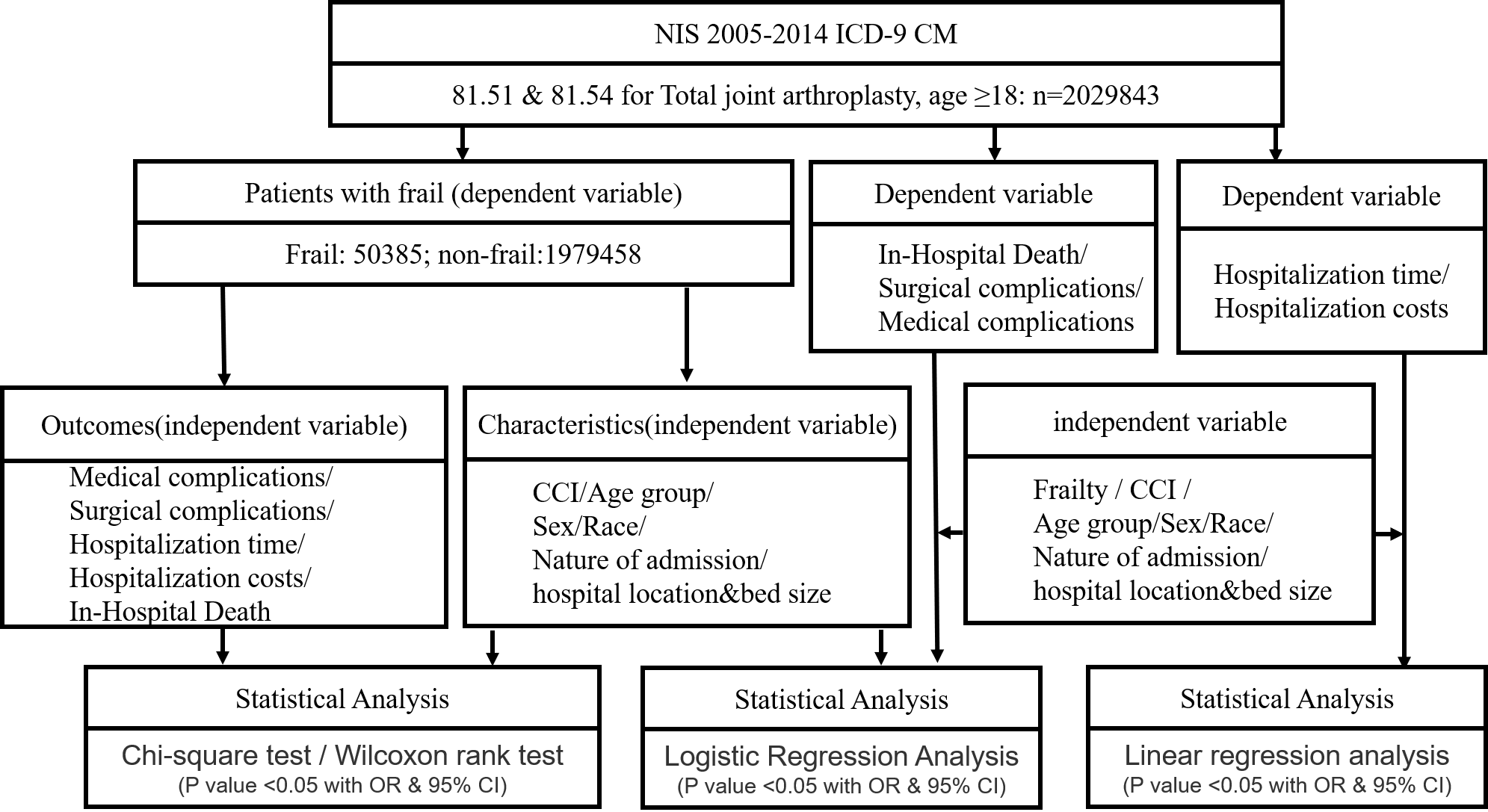

Supplement: Supplementary file 2 — Additional file 2: Supplementary figure 1. Flow diagram of the data collection and analysis. [file 12891_2022_5333_MOESM2_ESM.tif]
